# Functional Genetic Polymorphisms in the IL1RL1–IL18R1 Region Confer Risk for Ocular Behçet’s Disease in a Chinese Han Population

**DOI:** 10.3389/fgene.2020.00645

**Published:** 2020-07-03

**Authors:** Xiao Tan, Qingyun Zhou, Meng Lv, Handan Tan, Qingfeng Wang, Liming Zhang, Qingfeng Cao, Gangxiang Yuan, Guannan Su, Aize Kijlstra, Peizeng Yang

**Affiliations:** ^1^The First Affiliated Hospital of Chongqing Medical University, Chongqing Key Laboratory of Ophthalmology and Chongqing Eye Institute, Chongqing, China; ^2^University Eye Clinic Maastricht, Maastricht, Netherlands

**Keywords:** Behçet’s disease, uveitis, causal variant, bioinformatic analysis, functional study

## Abstract

Single nucleotide polymorphisms (SNPs) in the IL1RL1–IL18R1 region are associated with various immune-mediated diseases. This study was carried out to investigate the causal variant for ocular Behçet’s disease (BD) and elucidate its target genes in the IL1RL1–IL18R1 region. Nine candidate functional SNPs were prioritized with bioinformatics analysis, followed by a two-stage association study in 694 ocular BD patients and 1,458 unaffected controls. Functional studies were performed in the peripheral blood mononuclear cells (PBMCs) of 45 healthy men and 16 active male BD patients. Genotyping was performed using the MassARRAY System. The mRNA expressions of IL1RL1, IL18R1, IL18RAP, and SLC9A4 were assayed by real-time PCR and secretion of cytokines was examined by ELISA. Significantly lower frequencies of the rs12987977 GG genotype/G allele (*P*_c_ = 8.93 × 10^–7^, OR = 0.39; *P*_c_ = 2.60 × 10^–3^, OR = 0.77, respectively), rs12999364 TT genotype/T allele (*P*_c_ = 3.15 × 10^–4^, OR = 0.51; *P*_c_ = 1.13 × 10^–2^, OR = 0.80, respectively), and rs4851569 AA genotype/A allele (*P*_c_ = 3.29 × 10^–4^, OR = 0.52; *P*_c_ = 9.72 × 10^–3^, OR = 0.80, respectively) were observed in BD patients compared with the controls. Functional experiments revealed a downregulation of IL1RL1, IL18R1, and SLC9A4 and a decreased secretion of IFN-γ in the anti-CD3/CD28 antibody-treated PBMCs as well as a decreased production of TNF-α in the lipopolysaccharide (LPS)-stimulated PBMCs in carriers of the protective homozygous rs12987977/GG genotype compared with the TT genotype. Our findings show that functional SNPs—rs12987977, rs12999364, and rs4851569—in the IL1RL1–IL18R1 region confer susceptibility to ocular BD in a Chinese Han population. And IL1RL1, IL18R1, and SLC9A4 may be the target genes of rs12987977.

## Introduction

Behçet’s disease (BD) is a systemic autoinflammatory/autoimmune condition with multi-tissue manifestations, such as oral aphthae, genital ulcers, arthritis, and uveitis. Being one of the most common uveitis entities in China, it mainly affects young adults and has a chronic relapsing–remitting course (Yang et al., 2005). The exact etiology of BD remains largely unknown and is thought to be multifactorial, involving genetic, immunologic, and environmental factors (de Chambrun et al., 2012).

Genetic association studies are important since they may identify the pathological mechanisms causing BD and can be instrumental in the development of novel therapies. To date, HLA-B51 has been confirmed as the strongest genetic susceptibility factor for BD by several genome-wide association studies (GWAS) (Gul and Ohno, 2012; Kirino et al., 2013), although an increasing number of non-human leukocyte antigen (HLA) susceptibility variants have also been shown to be associated with this disease (Remmers et al., 2010; Lee et al., 2013). However, most susceptibility single nucleotide polymorphisms (SNPs) identified in GWAS were non-coding variants, and because of the incomplete coverage of SNPs in most GWAS and the phenomenon of co-inheritance of many variants, known as linkage disequilibrium (LD), functional studies addressing biological mechanisms which are aimed at pinning down the causal genetic variants and their gene targets in a given locus are still scarce (Gallagher and Chen-Plotkin, 2018). Fortunately, with the advent of public functional genomic databases such as GTEx (The GTEx Consortium, 2013), ENCODE (ENCODE Project Consortium, 2012), and Haploreg (Ward and Kellis, 2016), emerging studies are now reporting on this issue and promising results are obtained concerning the biological mechanisms that link susceptibility genetic variants with disease phenotypes (Allen et al., 2017; Cui et al., 2018).

An ongoing GWAS from our group suggested that the IL1RL1–IL18R1 region might harbor susceptibility loci for BD, although the *p*-values were below our GWAS threshold of 5 × 10^–8^ (unpublished data). It has been reported that sub-threshold SNPs could represent true disease risk loci that may have regulatory consequences and that an additional analysis of such SNPs may help to explain “missing heritability” when using a too stringent threshold (Wang et al., 2016). In addition, this region contains a cluster of immune-related genes that belong to the interleukin 1 receptor family (IL1RL2, IL1RL1, IL18R1, and IL18RAP). SNPs located in these genes were reported to be associated with various autoimmune or allergic diseases in earlier studies, such as inflammatory bowel disease and asthma (Liu et al., 2015; Zhu et al., 2018). Studies focusing on the biological function of the genes in this region also showed an important role in regulating the immune system. For instance, genetic ablation of IL1RL1 protected mice from developing experimental inflammatory bowel disease, and an antagonist of IL1RL1 could reduce the symptoms of colitis in these animals (Sedhom et al., 2013). The development of experimental autoimmune encephalomyelitis was attenuated in IL18R1-deficient mice (Gutcher et al., 2006). Taken together, this evidence highlights a crucial and pleiotropic role of this region in the development of immune-mediated diseases. The functional causal SNP and the exact susceptibility gene have, however, not yet been determined; therefore, despite the weak association in our GWAS, we decided to study this region in more detail in a large cohort of ocular BD patients and unaffected controls and also performed several functional experiments in peripheral blood mononuclear cells (PBMCs) from genotyped active BD patients and controls.

## Materials and Methods

### Ethical Considerations

This study was approved by the Clinical Ethical Committee of the First Affiliated Hospital of Chongqing Medical University (permit no. 2009-201008). The tenets of the Declaration of Helsinki were strictly followed in all procedures. Informed consent was collected from all subjects.

### Subjects

A total of 694 ocular BD patients and 1,458 ethnically matched unaffected Chinese Han volunteers independent of our ongoing GWAS cohorts (unpublished data) were recruited from the uveitis clinic in the Ophthalmology Department of the First Affiliated Hospital of Chongqing Medical University (Chongqing, China) (Yang et al., 2018). Patients were diagnosed following the criteria for the diagnosis of Behçet’s disease from the International Study Group for Behçet’s Disease (International Study Group for Behçet’s Disease, 1990). For the two-stage association study, the subjects were enrolled from 2008 to 2019. Another 45 healthy male volunteers and 16 active male ocular BD patients of Han ethnicity were also included for subsequent functional studies.

### Prioritization of Candidate Functional SNPs

The regional plot of GWAS susceptibility loci was generated by the Locuszoom website (LOCUSZOOM, RRID:SCR_009257). The plot of pairwise LD matrix analysis was created using LDlink^1^. Candidate functional SNPs that were in strong LD with the chosen index SNPs and their epigenomic annotations and transcription factor (TF) binding information were obtained *via* the Haploreg database (HaploReg, RRID:SCR_006796). Candidate target genes (protein coding) of functional SNPs with FDR ≤ 0.05 for SNP–gene pairs were queried from the GTEx portal (Genotype-Tissue Expression, RRID:SCR_013042) and 3DSNP databases^2^ (Lu et al., 2017).

### DNA Extraction and Genotyping

DNA was extracted from venous blood with the QIAamp DNA Blood Mini Kit (QIAGEN, Valencia, CA, United States) according to the manufacturer’s instructions. SNPs were genotyped with the MassARRAY system (Sequenom Inc., San Diego, CA, United States). The call rates of the SNPs tested in our study in the cases and controls were all above 95%.

### Cell Isolation and Culture

The PBMCs of 45 healthy male volunteers were isolated from fresh peripheral blood by Ficoll-Hypaque density gradient centrifugation, then cultured in 24-well plates with complete RPMI 1640 medium (consisting of 10% fetal bovine serum, 100 U/ml penicillin, and 100 μg/ml streptomycin) at a density of 2 × 10^6^ cells per well. The PBMCs of each individual were treated with 100 ng/ml lipopolysaccharide (LPS) (Sigma, MO, United States) for 1 day or a combination of anti-CD3 and anti-CD28 antibodies (5:1) (Miltenyi Biotec, Palo Alto, CA, United States) for 3 days, respectively, in an incubator with 5% CO_2_ at 37°C.

### Real-Time PCR

Total RNA from 45 healthy male volunteers was extracted with TRIzol reagent (Invitrogen, San Diego, CA, United States) from non-stimulated PBMCs, LPS-stimulated PBMCs, and anti-CD3/CD28 antibody-stimulated PBMCs, respectively. Prime Script RT reagent kit (TaKaRa, Dalian, China) was used for reverse transcription into cDNA. Relative mRNA expression assays were measured with the ABI 7500 Real-Time PCR System (ABI, Foster City, CA, United States) using appropriate primers of IL1RL1 (NM_016232.5), IL18R1 (NM_003855.5), IL18RAP (NM_003853.3), SLC9A4 (NM_001011552.4), and the reference gene β-actin (NM_001101.5) (Supplementary Table S1). The relative expression levels of genes were calculated with the 2^–ΔΔCt^ method. The representative dissociation curves of the PCR products are shown in Supplementary Figure S4.

### Enzyme-Linked Immunosorbent Assay

The concentrations of IL-1β, TNF-α, and IL-6 in the culture supernatants of the LPS-stimulated PBMCs as well as IFN-γ, IL-10, and IL-17 in the anti-CD3/CD28 antibody-stimulated PBMCs were quantified with the human Duoset enzyme-linked immunosorbent assay (ELISA) development kit (R&D Systems, Minneapolis, MN, United States) according to the instructions of the manufacturer. The representative standard curve of ELISA is shown in Supplementary Figure S5.

### Statistical Analysis

For genetic association analysis, the genotype and allele frequency data were analyzed using Typer4.0 software from the MassARRAY system. The Hardy–Weinberg equilibrium (HWE) of all tested SNPs in the controls was performed using the SHEsis online tool (SHEsis: Analysis Tools For Random Samples, RRID:SCR_002958) (Shi and He, 2005). Statistical power of sample size was calculated with the online tool of power and sample size calculator^3^. χ^2^ test, *P*-value, odds ratio (OR), 95% confidence intervals (95% CIs), and age- and sex-adjusted multivariate logistic regression analysis were calculated by SPSS (SPSS, RRID:SCR_002865). The *P*_c_ values were calculated from the original *P*-values in the χ^2^ test and logistic regression analysis with the Bonferroni correction in order to adjust for multiple comparisons. A correction factor of 9 was chosen, representing the number of SNPs analyzed in our study. The threshold of statistical significance of the *P*_c_ values was set as 0.05. For *in vitro* functional analysis, unpaired *t*-test or non-parametric Mann–Whitney test was used for comparisons between two independent groups, while one-way ANOVA or non-parametric Kruskal–Wallis test was chosen for comparisons among three independent groups according to different applicable conditions using GraphPad Prism 8 (GraphPad Prism, RRID:SCR_002798). Statistical significance was recognized as *P-*values lower than 0.05 (two-tailed).

### Data Availability Statement

The raw data supporting the conclusions of this manuscript will be made available by the authors, without undue reservation, to any qualified researcher upon request.

## Results

### Characteristics of the Study Subjects

The gender, age, and clinical characteristics of the 694 ocular BD patients and 1,458 unaffected controls are shown in Tables 1, 2. All BD patients had uveitis. The BD group consisted of 585 males (84.3%) and 109 females (15.7%), whereas 746 males (51.2%) and 712 (48.8%) females compose the control group. The proportion of females in the BD group is smaller than that of the control group (*P* < 0.05). The mean age of the BD group and the control group is 34.3 and 39.7 years, respectively (*P* < 0.05). Given the difference between these groups in terms of gender and age, we used multivariate logistic regression analysis to adjust for possible confounding effects.

**TABLE 1:** Demographic characteristics of Behçet’s disease (BD) patients and controls.

Demographic characteristics	BD (%)	Controls (%)	*P*-value
Females	109 (15.7)	712 (48.8)	<0.05
Males	585 (84.3)	746 (51.2)	
Mean age ± SD	34.3 ± 9.5	39.7 ± 10.1	<0.05

**TABLE 2:** Representative clinical features of patients with Behçet’s disease.

Clinical features	Total	%
Uveitis	694	100
Oral ulcer	694	100
Skin lesions	485	69.9
Genital ulcer	191	27.5
Arthritis	117	16.9
Pathergy reaction	52	7.5
Epididymitis	50	7.2
Perianal abscess	31	4.5
Thrombophlebitis	14	2.0
Gastrointestinal lesions	10	1.4

### Bioinformatics Analysis of Susceptibility Loci and Prioritization of Candidate Functional SNPs

**FIGURE 1:**
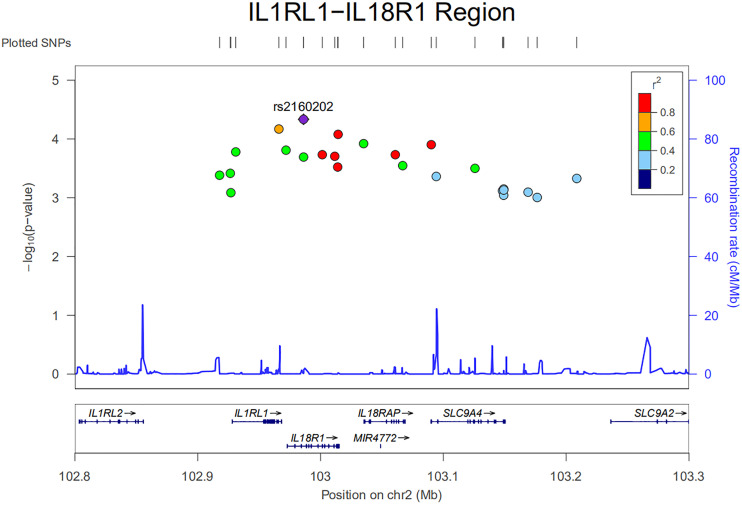
Regional plot of nominal susceptibility SNPs identified in our ongoing genome-wide association study (GWAS) in the IL1RL1–IL18R1 region.

**FIGURE 2:**
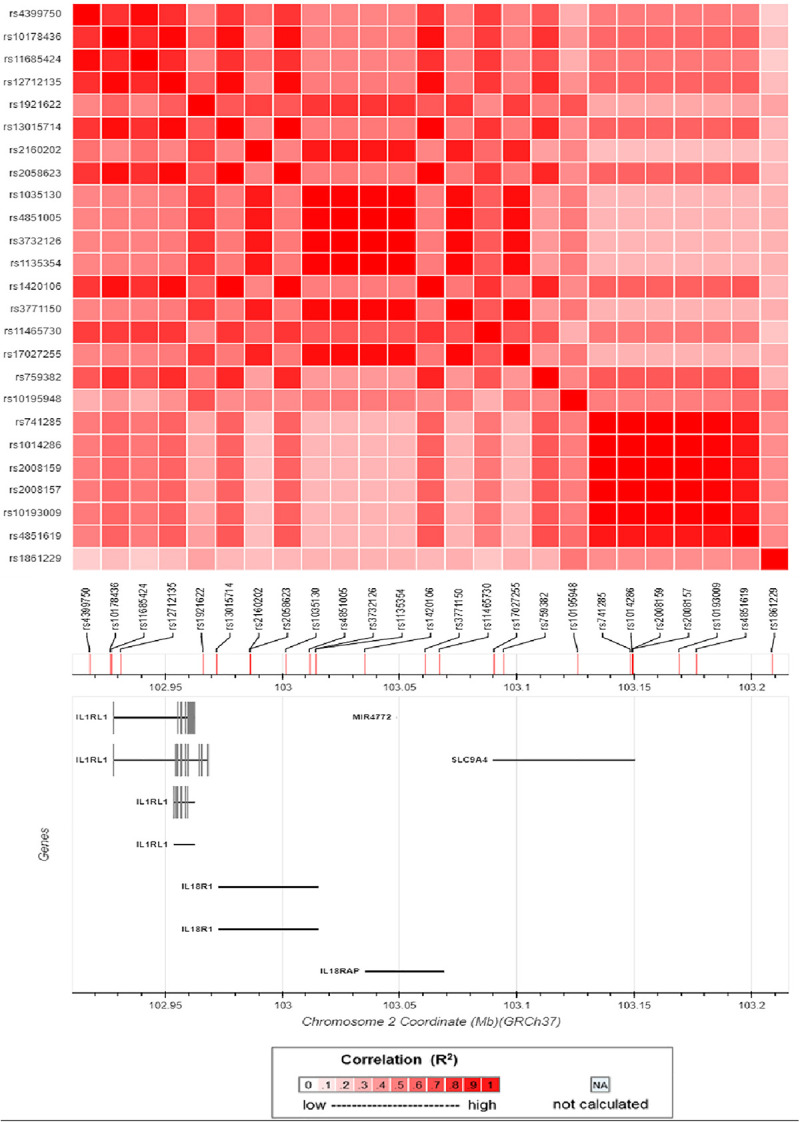
Matrix plot of pairwise linkage disequilibrium (LD) analysis of nominal susceptibility SNPs in the IL1RL1–IL18R1 region.

An ongoing Behçet’s disease GWAS in our laboratory identified a potential susceptibility region on chromosome 2q12, containing 25 non-coding SNPs with *P*-values below the GWAS threshold, but with a suggestive level of significance (4.63 × 10^–5^ < *P* < 9.88 × 10^–4^). These SNPs cover about 0.5 Mb on the chromosome and show a varying degree of LD with each other (Figure 1). They are located close to or in the genes encoding IL1RL2, ILIRL1, IL18R1, and IL18RAP (Figures 1, 2). Pairwise LD analysis revealed that rs2160202 and rs1420106 could capture almost all the other SNPs in this locus, and they were therefore chosen as the index SNPs for further bioinformatics analysis (Figure 2). The *r*^2^ between these two SNPs is 0.481.

It is now becoming apparent that causal variants that were thought to confer disease risk are not necessarily the tag variants detected by the microarrays used in GWAS, but variants that are in LD with the tag SNPs (Oldridge et al., 2015; Painter et al., 2016). Causal SNPs often reside in regulatory elements (promoter and enhancer) and could bind to certain TFs (Painter et al., 2016). They influence the disease susceptibility partly by regulating the expressions of its target genes in disease-relevant tissues and cell types (Farh et al., 2015). In addition, by long-range chromosome interactions, causal variants could even regulate distal gene targets. Thus, all SNPs that were in strong LD (*r*^2^ > 0.8) with these two index SNPs were analyzed using genomic structure data from the Han Chinese in Beijing (CHB) and Southern Han Chinese (CHS) databank^4^. Candidate functional SNPs were prioritized according to the following criteria: (1) whether they were expression quantitative trait loci (eQTLs) in the GTEx portal; (2) whether they had three-dimensional interacting genes in the 3DSNP database; (3) whether they were located in regulatory elements (promoter/enhancer) in immune cells with typical histone acetylation markers such as H3K27ac, H3K4Me1, and H3K4Me3; and (4) whether they were occupied by TFs or could change the binding motif of certain TFs in the Haploreg database. With the criteria mentioned above, we prioritized nine candidate functional SNPs for further association studies (Table 3).

**TABLE 3:** Prioritization of nine candidate functional variants in susceptibility to Behçet’s disease (BD) in the IL1RL1–IL18R1 region.

Candidate SNPs	Location	eQTL genes and 3D interacting genes^a^	Regulatory elements	TFs
rs2160202	Intron of IL18R1	IL1RL1, SLC9A4, IL18RAP, IL18R1, IL1RL2	Enhancer-like region	TBX5
rs4851569	Intron of IL18R1	IL1RL1, IL18R1, IL18RAP, SLC9A4	Enhancer-like region	IRF4, PU1, PAX5N19
rs12999364	Intron of IL18R1	IL1RL1, IL18R1, SLC9A4, IL18RAP	Enhancer-like region	EBF1, POL24H8
rs12987977	Intron of IL18R1	IL1RL1, IL18R1, SLC9A4, IL18RAP	Enhancer-like region	CTCF
rs1420106	104 bp 5′ of IL18RAP	IL1RL1, IL18R1, SLC9A4, IL18RAP	Promoter-like region	JUN, MYC, STAT1
rs3755267	Intron of IL18RAP	IL1RL1, IL18R1, SLC9A4, IL18RAP	Enhancer-like region	MYC, CTCF, PU1
rs6746271	Intron of IL18RAP	IL1RL1, IL18R1, SLC9A4, IL18RAP	Enhancer-like region	NFKB, IRF4
rs2058660	Intron of IL18RAP	IL1RL1, IL18R1, SLC9A4, IL18RAP	Enhancer-like region	RXRA, HNF4
rs917997	1.5 kb 3′ of IL18RAP	IL1RL1, IL18R1, SLC9A4, IL18RAP	Enhancer-like region	TAL1

*^a^For detailed information concerning P-values and effect size of eQTLs (SNPs) in different tissues, please visit the GTEx portal (https://www.gtexportal.org/home/).*

### Two-Stage Association Study

To confirm the association with the ocular BD phenotype and fine-map the true genetic signal in the IL1RL1–IL18R1 region, we next performed a two-stage association study for the nine candidate functional SNPs with 694 BD patients and 1,458 unaffected controls that were independent of our GWAS groups. None of the tested SNPs deviated from the HWE. A χ^2^ test in the first-stage analysis, where 434 BD patients and 558 controls were included, showed significantly decreased frequencies of the rs12987977 (NC_000002.11:g.102975336T > G) GG genotype/G allele (*P*_c_ = 2.30 × 10^–5^, OR = 0.32; *P*_c_ = 2.88 × 10^–4^, OR = 0.66, respectively), rs12999364 (NC_000002.11:g.102974129C > T) TT genotype/T allele (*P*_c_ = 4.76 × 10^–3^, OR = 0.46; *P*_c_ = 2.30 × 10^–3^, OR = 0.70, respectively), and rs4851569 (NC_000002.11:g.102983247C > A) AA genotype/A allele (*P*_c_ = 3.14 × 10^–3^, OR = 0.46; *P*_c_ = 2.12 × 10^–3^, OR = 0.70, respectively) in the BD group compared to the controls (Table 4). We were not able to detect an association with ocular BD for the other six SNPs (Supplementary Table S2), and these SNPs were not investigated in our further studies.

**TABLE 4:** Association of three SNPs with ocular Behçet’s disease (BD).

SNPs	Stage	Genotype/allele	Cases		Controls		*P*-value	*P*_c_ value	OR (95% CI)
			*N*	%	*N*	%			
rs12987977	First stage	GG	21	5.0	78	14.2	2.56 × 10^–6^	2.30 × 10^–5^	0.32 (0.19–0.52)
		GT	199	47.2	257	46.7	0.89	NS	1.02 (0.79–1.31)
		TT	202	47.9	215	39.0	6.14 × 10^–3^	NS	1.43 (1.11–1.85)
		G	241	28.6	413	37.5	3.20 × 10^–5^	2.88 × 10^–4^	0.66 (0.55–0.81)
		T	603	71.4	687	62.5	3.20 × 10^–5^	2.88 × 10^–4^	1.50 (1.24–1.82)
	Second stage	GG	18	7.3	118	13.4	9.20 × 10^–3^	NS	0.51 (0.30–0.85)
		GT	127	51.4	378	42.9	1.74 × 10^–2^	NS	1.41 (1.06–1.87)
		TT	102	41.3	385	43.7	0.50	NS	0.91 (0.68–1.21)
		G	163	33.0	614	34.8	0.44	NS	0.92 (0.75–1.14)
		T	331	67.0	1,148	65.2	0.44	NS	1.09 (0.88–1.34)
	Combined	GG	39	5.8	196	13.7	9.92 × 10^–8^	8.93 × 10^–7^	0.39 (0.27–0.56)
		GT	326	48.7	635	44.4	6.20 × 10^–2^	NS	1.19 (0.99–1.43)
		TT	304	45.4	600	41.9	0.13	NS	1.15 (0.96–1.39)
		G	404	30.2	1,027	35.9	2.89 × 10^–4^	2.60 × 10^–3^	0.77 (0.67–0.89)
		T	934	69.8	1,835	64.1	2.89 × 10^–4^	2.60 × 10^–3^	1.29 (1.13–1.49)
rs12999364	First stage	CC	201	47.9	211	39.0	5.93 × 10^–3^	NS	1.44 (1.11–1.86)
		CT	189	45.0	253	46.8	0.59	NS	0.93 (0.72–1.20)
		TT	30	7.1	77	14.2	5.29 × 10^–4^	4.76 × 10^–3^	0.46 (0.30–0.72)
		C	591	70.4	675	62.4	2.56 × 10^–4^	2.30 × 10^–3^	1.43 (1.18–1.73)
		T	249	29.6	407	37.6	2.56 × 10^–4^	2.30 × 10^–3^	0.70 (0.58–0.85)
	Second stage	CC	107	41.8	380	43.2	0.68	NS	0.94 (0.71–1.25)
		CT	127	49.6	378	43.0	6.13 × 10^–2^	NS	1.30 (0.99–1.72)
		TT	22	8.6	121	13.8	2.82 × 10^–2^	NS	0.59 (0.37–0.95)
		C	341	66.7	1,138	64.7	0.44	NS	1.09 (0.88–1.34)
		T	171	33.4	620	35.3	0.44	NS	0.92 (0.75–1.13)
	Combined	CC	308	45.5	591	41.6	9.36 × 10^–2^	NS	1.17 (0.97–1.41)
		CT	317	46.8	631	44.4	0.30	NS	1.10 (0.92–1.32)
		TT	52	7.7	198	13.9	3.50 × 10^–5^	3.15 × 10^–4^	0.51 (0.37–0.71)
		C	933	68.9	1,813	63.8	1.25 × 10^–3^	1.13 × 10^–2^	1.26 (1.09–1.44)
		T	421	31.1	1,027	36.2	1.25 × 10^–3^	1.13 × 10^–2^	0.80 (0.69–0.91)
rs4851569	First stage	CC	200	46.5	209	37.9	6.38 × 10^–3^	NS	1.43 (1.10–1.84)
		CA	199	46.3	263	47.6	0.67	NS	0.95 (0.74–1.22)
		AA	31	7.2	80	14.5	3.49 × 10^–4^	3.14 × 10^–3^	0.46 (0.30–0.71)
		C	599	69.7	681	61.7	2.36 × 10^–4^	2.12 × 10^–3^	1.43 (1.18–1.72)
		A	261	30.3	423	38.3	2.36 × 10^–4^	2.12 × 10^–3^	0.70 (0.58–0.85)
	Second stage	CC	109	42.6	380	43.1	0.88	NS	0.98 (0.74–1.30)
		CA	125	48.8	381	43.2	0.11	NS	1.25 (0.95–1.66)
		AA	22	8.6	120	13.6	3.22 × 10^–2^	NS	0.60 (0.37–0.96)
		C	343	67.0	1,141	64.8	0.35	NS	1.11 (0.90–1.36)
		A	169	33.0	621	35.2	0.35	NS	0.91 (0.73–1.12)
	Combined	CC	309	45.1	589	41.1	8.09 × 10^–2^	NS	1.18 (0.98–1.41)
		CA	323	47.2	644	44.9	0.34	NS	1.09 (0.91–1.31)
		AA	53	7.7	200	14.0	3.65 × 10^–5^	3.29 × 10^–4^	0.52 (0.38–0.71)
		C	941	68.7	1,822	63.6	1.08 × 10^–3^	9.72 × 10^–3^	1.26 (1.10–1.44)
		A	429	31.3	1,044	36.4	1.08 × 10^–3^	9.72 × 10^–3^	0.80 (0.69–0.91)

**SNP, single nucleotide polymorphism; P_c_, Bonferroni-corrected P-value; NS, not significant; OR, odds ratio; 95% CI, 95% confidence interval.**

To expand the sample size in the first-stage study, another independent cohort consisting of 260 ocular BD patients and 900 unaffected controls was added to the association study. The results in the second-stage study also showed suggestive lower frequencies of the rs12987977/GG genotype (*P* = 9.20 × 10^–3^, OR = 0.51), rs12999364/TT genotype (*P* = 2.82 × 10^–2^, OR = 0.59), and the rs4851569/AA genotype (*P* = 3.22 × 10^–2^, OR = 0.60) before multiple corrections in BD compared to the controls (Table 4). After combining the two stages together, it was confirmed that BD patients had lower frequencies of the rs12987977 GG genotype/G allele (*P*_c_ = 8.93 × 10^–7^, OR = 0.39; *P*_c_ = 2.60 × 10^–3^, OR = 0.77, respectively), rs12999364 TT genotype/T allele (*P*_c_ = 3.15 × 10^–4^, OR = 0.51; *P*_c_ = 1.13 × 10^–2^, OR = 0.80, respectively), and the rs4851569 AA genotype/A allele (*P*_c_ = 3.29 × 10^–4^, OR = 0.52; *P*_c_ = 9.72 × 10^–3^, OR = 0.80, respectively) in comparison with the controls (Table 4). We also calculated the statistical power for our combined sample size. The power to detect the difference in genotypic frequency between the BD and control groups for all the three susceptibility SNPs is more than 90%.

To adjust for possible confounding effects of gender and age, a multivariate logistic regression analysis of various genetic models was performed for the three susceptibility SNPs. The results of the co-dominant model were in agreement with the previous χ^2^ tests. In addition, it also suggested that the associations of the rs12999364–T allele, rs12987977–G allele, and the rs4851569–A allele with BD behaved as recessive models, respectively (Table 5).

**TABLE 5:** Age- and sex-adjusted multivariate logistic regression analysis of the risk of ocular Behçet’s disease (BD) with three susceptibility SNPs in co-dominant, dominant, and recessive models.

Model		Cases		Controls		Multivariate logistic regression		
		*N*	%	*N*	%	*P-*value	*P*_c_ value	OR (95% CI)
**rs12999364**								
Co-dominant	CC	308	45.5	591	41.6	Ref.	Ref.	
	CT	317	46.8	631	44.4	0.91	NS	1.01 (0.82–1.25)
	TT	52	7.7	198	13.9	1.00 × 10^–3^	9.00 × 10^–3^	0.55 (0.39–0.79)
Dominant	CC	308	45.5	591	41.6	Ref.	Ref.	
	CT + TT	369	54.5	829	58.4	0.33	NS	0.91 (0.74–1.11)
Recessive	CC + CT	625	92.3	1,222	86.1	Ref.	Ref.	
	TT	52	7.7	198	13.9	1.00 × 10^–3^	9.00 × 10^–3^	0.55 (0.39–0.78)
**rs12987977**								
Co-dominant	TT	304	45.4	600	41.9	Ref.	Ref.	
	GT	326	48.7	635	44.4	0.59	NS	1.06 (0.86–1.31)
	GG	39	5.8	196	13.7	5.30 × 10^–5^	4.77 × 10^–4^	0.44 (0.30–0.66)
Dominant	TT	304	45.4	600	41.9	Ref.	Ref.	
	GT + GG	365	54.6	831	58.1	0.42	NS	0.92 (0.75–1.13)
Recessive	TT + GT	630	94.2	1,235	86.3	Ref.	Ref.	
	GG	39	5.8	196	13.7	1.30 × 10^–5^	1.17 × 10^–4^	0.43 (0.30–0.63)
**rs4851569**								
Co-dominant	CC	309	45.1	589	41.1	Ref.	Ref.	
	CA	323	47.2	644	44.9	0.88	NS	1.02 (0.82–1.25)
	AA	53	7.7	200	14.0	2.00 × 10^–3^	1.80 × 10^–2^	0.57 (0.40–0.81)
Dominant	CC	309	45.1	589	41.1	Ref.	Ref.	
	CA + AA	376	54.9	844	58.9	0.38	NS	0.91 (0.75–1.12)
Recessive	CC + CA	632	92.3	1,233	86.0	Ref.	Ref.	
	AA	53	7.7	200	14.0	1.00 × 10^–3^	9.00 × 10^–3^	0.56 (0.40–0.79)

**The hypothesis test was performed using the Wald χ^2^ test*.*

### Effect of rs12987977 on IL1RL1, IL18R1, IL18RAP, and SLC9A4 Expression

**FIGURE 3:**
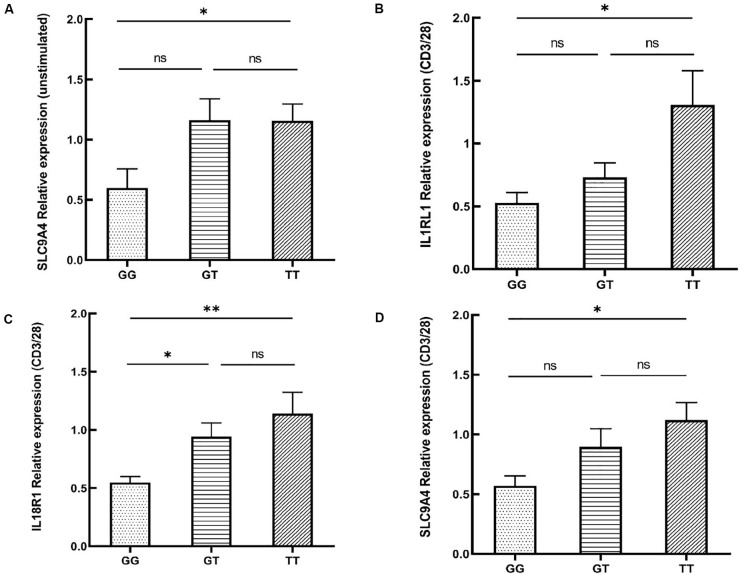
Significant differential expressions of candidate target genes between various genotypes of rs12987977 in non-stimulated peripheral blood mononuclear cells (PBMCs) **(A)** and anti-CD3/CD28-stimulated PBMCs **(B–D)** from healthy male controls (GG = 10, GT = 17, TT = 18). Data are shown as the mean ± SEM. ^∗^*P* < 0.05; ^∗∗^*P* < 0.01.

rs12987977 showed the strongest association with BD and was therefore chosen for further investigations on possible causal biological mechanisms. Previous bioinformatics analysis with the 3DSNP database implied that rs12987977 could interact with IL1RL1, IL18R1, and IL18RAP in the 3D chromosome. Analysis with the GTEx portal tool also indicated that it correlated with the expressions of IL1RL1, IL18R1, IL18RAP, and SLC9A4 in different cell types and tissues. The GTEx portal lacks data for PBMCs or any other specific immune cells that are considered relevant to autoimmune disease. Donors in the GTEx project were of various ancestries. To investigate the target gene(s) of rs12987977 that might play an important role in the development of BD in Chinese Han, we performed a real-time quantitative PCR experiment to determine the effect of the different genotypes of rs12987977 on the expressions of those candidate target genes in the PBMCs obtained from a newly recruited group of genotyped healthy individuals. To rule out a possible confounding effect of gender, all the 45 volunteers enrolled for these functional studies are men. The role of various stimuli was tested and included unstimulated control, LPS-stimulated (non-specific inflammation signal), and anti-CD3/CD28 antibody-stimulated (antigen-mimicking signal). The results showed that, in unstimulated cells, there was a slightly significant downregulation of the mRNA level of SLC9A4 in rs12987977/GG carriers compared with TT carriers (*P* = 0.044; Figure 3A). There was no significant difference between the different genotypes for the expressions of the other three genes in the unstimulated controls (Supplementary Figures S1A–C). When the PBMCs were stimulated with the anti-CD3/CD28 antibody, the expressions of three out of four genes tested showed a significant difference between the different genotypes (Figures 3B–D), while no significant difference was seen between the different genotypes and the expression of IL18RAP (Supplementary Figure S1D). A significantly lower expression of IL1RL1 in rs12987977/GG carriers was observed as compared to TT carriers (*P* = 0.021; Figure 3B), and a significantly lower expression of IL18R1 in rs12987977/GG carriers in contrast to the GT/TT carriers (^∗^*P* = 0.022 and ^∗∗^*P* = 0.002, respectively; Figure 3C). Similar to the findings in non-stimulated PBMCs, the expression of SLC9A4 in rs12987977/GG carriers was significantly decreased compared with the TT carriers following the anti-CD3/CD28 antibody stimulation (*P* = 0.018; Figure 3D). When stimulated with LPS, none of the genes tested showed a significant effect of genotype on the mRNA expression (Supplementary Figures S1E–H).

### Differential Expression Analysis Between Active BD Patients and Healthy Controls

To investigate whether the expressions of the target genes of rs12987977 were dependent on the disease state, we performed a real-time quantitative PCR assay in the PBMCs of 16 active BD patients and 45 healthy controls. All the patients and controls tested are men and individuals of the same genotype were compared. No differences were observed between the patients and the controls (Supplementary Figures S2A–H).

### Effect of rs12987977 on Cytokine Production

**FIGURE 4:**
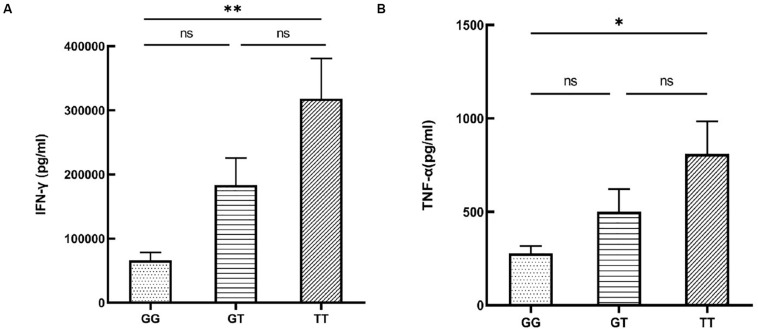
The influence of various rs12987977 genotypes on the secretion of IFN-γ in anti-CD3/CD28-stimulated peripheral blood mononuclear cells (PBMCs) **(A)** and TNF-α in lipopolysaccharide (LPS)-stimulated PBMCs **(B)** (GG = 9, GT = 17, TT = 18). Data are shown as the mean ± SEM. ^∗^*P* < 0.05; ^∗∗^*P* < 0.01.

To investigate whether the functional SNP could indirectly affect disease risk by exerting an influence on the release of pro-inflammatory or anti-inflammatory cytokines that had been well-studied in autoimmune diseases, we tested cytokine secretion by PBMCs from healthy male volunteers with various genotypes of rs12987977. We failed to collect the cell culture supernatant of one subject by accident. Individuals carrying the TT genotype had an elevated secretion of IFN-γ (*P* = 0.002; Figure 4A) when stimulated with anti-CD3/CD28 and an increased TNF-α (*P* = 0.021; Figure 4B) production when stimulated with LPS as compared to those with the GG genotype. No effect of genotype was observed on the secretion of IL-17, IL-10, IL-1β, or IL-6 (Supplementary Figures S3A–D).

## Discussion

In the current study, we identified nine candidate functional variants in the IL1RL1–IL18R1 region on chromosome 2 using public bioinformatics tools. Significant associations with ocular BD were found in three of these SNPs in a combined two-stage association study, including rs12987977, rs12999364, and rs4851569. The second-stage study by itself lacked statistically significant results, which may be due to the fact that the sample size of BD patients in the second stage was too small to provide sufficient statistical power. These three SNPs are novel susceptibility variants for BD. Since rs12987977 showed the strongest association with BD, we focused on this genetic variant to study the effect of genotype on the causal biological mechanisms. *In vitro* allele-specific expression analysis showed that various genotypes of the susceptibility SNP rs12987977 were correlated with the mRNA expressions of IL1RL1, IL18R1, and SLC9A4. Significant downregulation of the SLC9A4 mRNA level was observed in unstimulated PBMCs along with IL1RL1 and SLC9A4 in the anti-CD3/CD28 antibody-stimulated PBMCs of rs12987977/GG carriers compared with the TT carriers. The results also showed a significant downregulation of IL18R1 in rs12987977/GG carriers compared with the GT and TT carriers. Furthermore, cytokine production analysis between various genotypes of rs12987977 indicated significantly lower secretions of IFN-γ in the anti-CD3/CD28 antibody-stimulated PBMCs and TNF-α in the LPS-stimulated PBMCs of rs12987977/GG carriers compared with the TT carriers. These findings are in agreement with the fact that the TT genotype is the risk genotype for developing BD.

The SNP rs4851569 discovered in our study is a novel genetic variant, and to our knowledge, no disease association has been reported for this SNP. SNP rs12987977 has been reported to be associated with both Crohn’s disease and ulcerative colitis in a European population (Ellinghaus et al., 2016). The BD susceptibility variant rs12999364 found in our study was also shown to confer risk of asthma in a Dutch cohort (Reijmerink, 2008). Two SNPs (rs1420098 and rs1420101) that are in strong LD (*r*^2^ > 0.8) with rs12987977 were reported to be susceptibility factors for IBD and asthma in other studies (Liu et al., 2015; Demenais et al., 2018). The evidence indicates a pleiotropic role for this region in the development of immune-mediated diseases. Chromosome 2q12, which contains the IL1RL1–IL18R1 region, harbors several genes of the interleukin 1 receptor family (IL1RL2, IL1RL1, IL18R1, and IL18RAP). IL1RL1 (also known as ST2) was identified as a target gene of rs12987977 in our study and is a receptor of interleukin-33 (IL-33) (De la Fuente et al., 2015). It is expressed in various cell types, including dendritic cells, T helper 1 (Th1) cells, T helper 2 (Th2) cells, CD8^+^ T cells, NK cells, B cells, and neutrophils (De la Fuente et al., 2015; Cayrol and Girard, 2018). The function of the IL-33/ST2 axis has been investigated in asthma, suggesting an important role in allergic disease (Grotenboer et al., 2013; Snelgrove et al., 2014). Nevertheless, emerging evidence suggests that this signaling pathway also plays an important role in chronic inflammatory diseases (Verri et al., 2010; Li et al., 2015). Increased expressions of IL-33 and ST2 have been observed in skin lesions of vitiligo patients (Li et al., 2015). Administration of a blocking anti-ST2 antibody at the onset of collagen-induced arthritis attenuated the severity of the disease in mice and reduced joint destruction and was associated with a marked decrease in IFN-γ production by draining lymph node cells (Palmer et al., 2009). In a mouse model of methylated bovine serum albumin (mBSA)-induced arthritis, suppression of IL-33R (IL1RL1) expression in neutrophils could prevent IL-33-mediated neutrophil migration into the knee joint (Verri et al., 2010). Besides IL1RL1, we showed that rs12987977 also targets IL18R1. The latter forms a receptor complex with IL18RAP, responsible for the binding of the pro-inflammatory cytokine IL-18 (Yasuda et al., 2019). It can mediate IFN-γ synthesis from Th1 cells and contributes to Th1 differentiation (Tominaga et al., 2000). An increased IL18R1 mRNA expression has been shown in both cerebrospinal fluid cells and PBMCs in multiple sclerosis (Gillett et al., 2010). Blocking IL18R1 in MRL-Fas^lpr^ mice, which develop a systemic lupus erythematosus-like disease, results in significant reductions in renal pathology and the IFN-γ and TNF-α mRNA levels (Kinoshita et al., 2004). Further studies showed that deletion of IL-18R1 in intestinal epithelial cells inhibited mucosal damage and colitis in a mouse model of ulcerative colitis (Nowarski et al., 2015). The third gene that we identified as a target for rs12987977 is SLC9A4 (Solute Carrier Family 9 Member A4). SLC9A4 is a sodium/hydrogen exchanger involved in intracellular pH regulation and cell volume (Beltrán et al., 2008; Arena et al., 2012). It also functions as a transporter of glucose, bile salts, organic acids, and amine compounds (Beltrán et al., 2008; Arena et al., 2012). Its role in the pathogenesis of immune-mediated diseases has, to our knowledge, not yet been reported, although recent studies have pointed out a role of basic molecules in T cell differentiation (Mcgaha et al., 2012). Taken together, the evidence suggests a pro-inflammatory role for IL1RL1 and IL18R1 in autoimmune disease. The genetic control of these genes by rs12987977 is supported by the observation that individuals with the protective homozygous GG genotype of rs12987977 had significantly lower IL1RL1 and IL18R1 mRNA expressions and decreased secretions of IFN-γ and TNF-α from their PBMCs treated with certain extracellular stimuli. This is in line with the hypothesis that certain environmental factors are needed to trigger the development of BD in a genetically predisposed individual, whereby a multiplicity of genes, all leading to a pro-inflammatory state, may eventually lead to a full-blown disease. Until now, we were not able to detect differential expressions for IL1RL1, IL18R1, and SLC9A4 when comparing active BD patients and controls carrying the same genotype. There are a number of explanations for this observation. We investigated whether the target genes of rs12987977 (IL1RL1, IL18R1, and SLC9A4) were differentially expressed between BD and controls using 16 active BD patients and 45 controls. For the controls, we planned to recruit at least 10 cases for each of the three genotypes. After we collected 45 controls and genotyped them, we found that we had sufficient samples from each genotype. However, it was much more difficult to collect the same number of active BD patients during the same time period because most BD patients had already consulted other hospitals and had the inflammation under control before they came to us and could therefore not be included in the “active” BD group. The unequal sample size may therefore be one reason, and another possible reason is that most active BD patients were under immunosuppressive medication, which might affect the expressions of these genes.

It has been reported that patients with BD have a lower incidence of allergic diseases, such as atopic dermatitis, allergic rhinitis, bronchial asthma, and food/drug allergies, than the entire population in Japan (Horie et al., 2016). In our study, none of the controls had allergic disease and we only recorded a self-reported atopic dermatitis in 89 of our BD patients. A stratification analysis in BD cases with atopic dermatitis did not reveal a significant genetic correlation, which may be due to the fact that the numbers were too small to perform a meaningful genetic association analysis (data not shown). Future research with more complete information concerning the presence of allergic diseases in BD is required to adequately address this issue.

In our current study, we only used GTEx and 3DSNP to find the candidate target genes of rs12987977 and focused on protein-coding genes. Other databases may also provide useful information. For instance, rs12987977 has been shown to be associated with the expressions of AC007278.3, AC007278.2, IL1RL1, IL18R1, and MFSD9 in the whole blood and/or PBMCs of Europeans (see website of eQTLGen^5^) (Võsa et al., 2018). In future studies, we would like to investigate whether rs12987977 is also associated with the expressions of other genes (including non-coding genes) using more databases.

How an intron variant of IL18R1 such as rs12987977 affects the expressions of the target genes mentioned above is not yet clear. Analysis with bioinformatics tools suggests that it resides in the enhancer region of various cells of the immune system, including T cells, B cells, monocytes, natural killer cells, and neutrophils, and could bind to a well-studied transcription factor named CTCF that has been reported to be instrumental in chromosome looping (Tang et al., 2015; Nora et al., 2017). We suggest that the susceptibility variant rs12987977 may regulate the expressions of these gene targets by possibly changing the activity of the enhancer region, affecting chromosome interactions, or by altering the affinity of binding to CTCF when shifted from the T allele to the G allele. However, more functional studies should be carried out to clarify these possible mechanisms.

An earlier study of our group has identified rs2058660 as a susceptibility genetic variant for ocular BD in a Chinese Han cohort with G as the risk allele (*P*_c_ = 5.94 × 10^–6^, OR = 1.33) in a total of 1,063 patients and 1,872 controls that did not exclude the GWAS cohorts (Tan et al., 2016). In the current study, this SNP showed a weak association with BD whereby the G allele was also identified as the risk factor (*P* = 0.047, OR = 1.198) in the first-stage association study, but the significance disappeared after Bonferroni correction for multiple comparisons. The samples used in the first stage of these two studies are different, and the sample size was also not the same. The difference in sampling could partly account for this discrepancy. Therefore, we cannot entirely exclude the possibility that the SNP rs2058660 may also be associated with ocular BD when this is tested in a larger cohort.

There are several limitations to our study. Firstly, our BD patients were recruited from an ophthalmology department and all had uveitis. However, not all patients with BD necessarily have uveitis, and they may consult different medical departments depending on their main complaints. It would therefore be very interesting to see whether our findings concerning the associations found in the IL1RL1–IL18R1 region confer susceptibility to Behçet’s disease itself or to the development of ocular lesions in this disease. Future studies including BD patients from other medical departments (e.g., rheumatology) and other subpopulations are required to address this issue. Secondly, our results showed a correlation between various genotypes of rs12987977 and the expressions of candidate susceptibility genes and predicted the biological mechanisms underlying this correlation with bioinformatic methods, but further confirmation studies using enhancer reporter assays, CHIP-seq, and chromosome conformation capture should be carried out to elucidate the exact mechanism. Third, due to economic reasons, we did not expand the sample size for those SNPs that showed no association with BD after stringent corrections in the first-stage study. Larger cohorts are needed to study the association with BD for these SNPs in future studies. Lastly, we used primers for total isoforms for each gene instead of separate splice variants. More detailed studies regarding certain gene isoforms should be performed in the future to help us understand the function of the various gene isoforms.

## Conclusion

Our findings show that several functional SNPs—rs12987977, rs12999364, and rs4851569—residing in the IL1RL1–IL18R1 region confer susceptibility to ocular BD in a Chinese Han population. We focused on rs12987977 and showed that it targets IL1RL1, IL18R1, and SLC9A4. Further studies are needed to reveal the exact mechanism whereby this variant affects the expressions of these target genes and how this eventually may lead to the development of ocular BD.

## Data Availability Statement

The associations of the three susceptibility SNPs with Behcet’s disease from our manuscript are available at the ClinVar database of NCBI (accession numbers: SCV001334435 - SCV001334437).

## Ethics Statement

The studies involving human participants were reviewed and approved by the Clinical Ethical Committee of the First Affiliated Hospital of Chongqing Medical University (permit no. 2009-201008). Written informed consent to participate in this study was provided by the participants’ legal guardian/next of kin.

## Author Contributions

XT conceptualized and designed the study, carried out the analyses, drafted the initial manuscript, and revised the manuscript. QZ and ML designed the study, collected data, and reviewed the manuscript. HT, QW, and LZ reviewed the manuscript for important intellectual content. QC and GY collected samples and reviewed the manuscript. GS, AK, and PY reviewed the manuscript for important intellectual content and revised it. All authors approved the final manuscript as submitted and agreed to be accountable for all aspects of the work.

## Conflict of Interest

The authors declare that the research was conducted in the absence of any commercial or financial relationships that could be construed as a potential conflict of interest.

## Footnotes

1 https://ldlink.nci.nih.gov/

2 http://cbportal.org/3dsnp/

3 http://powerandsamplesize.com/

4 https://www.ncbi.nlm.nih.gov/variation/tools/1000genomes/

5 http://www.eqtlgen.org/cis-eqtls.html
