# Supplementary figures and images for: Functional Genetic Polymorphisms in the IL1RL1–IL18R1 Region Confer Risk for Ocular Behçet’s Disease in a Chinese Han Population
Source: Front Genet. 2020 Jul 3;11:645. doi: 10.3389/fgene.2020.00645 (PMC7350896; doi:10.3389/fgene.2020.00645)

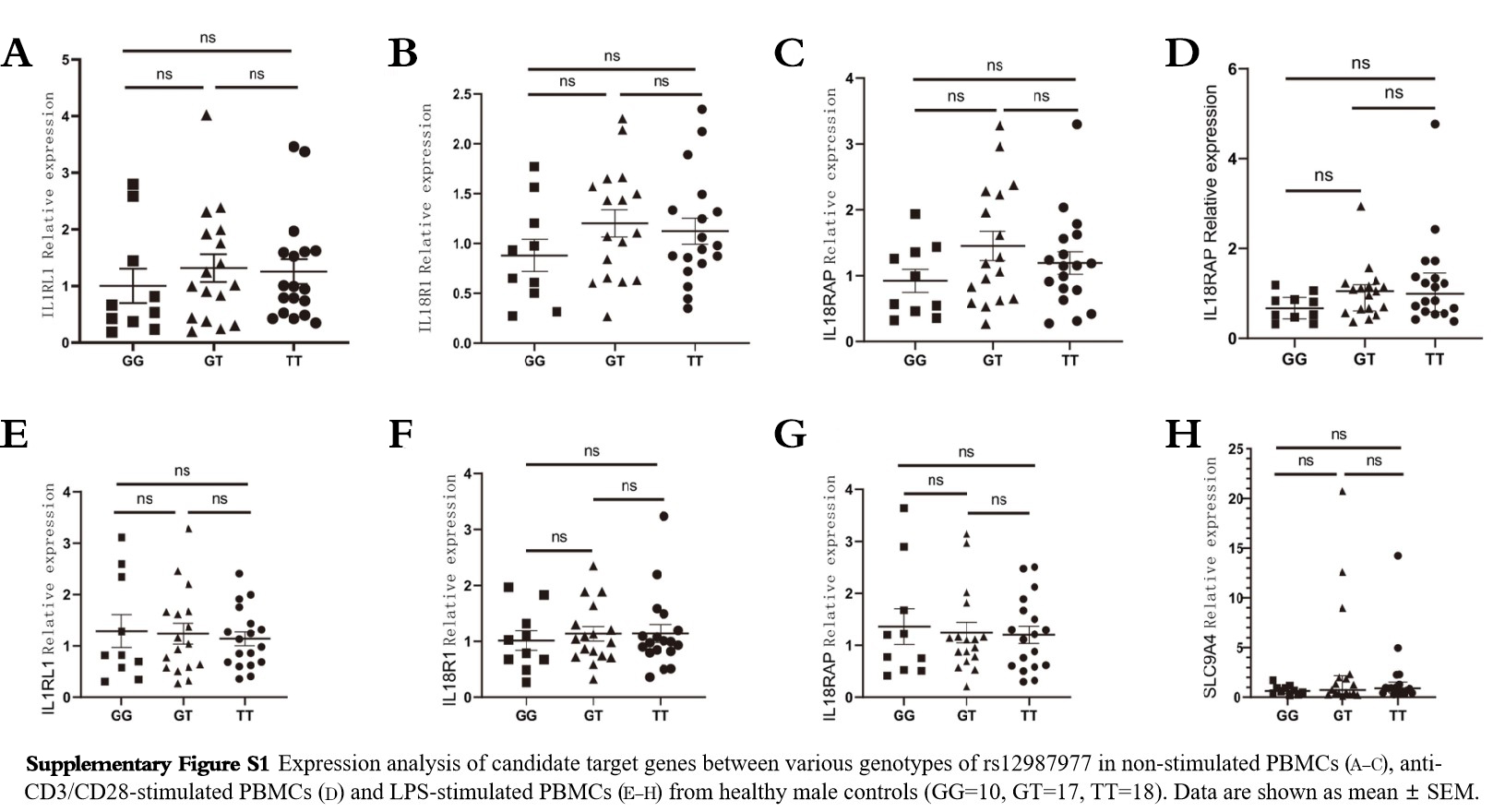

Supplement: Supplementary file 1 [file Image_1.jpg]

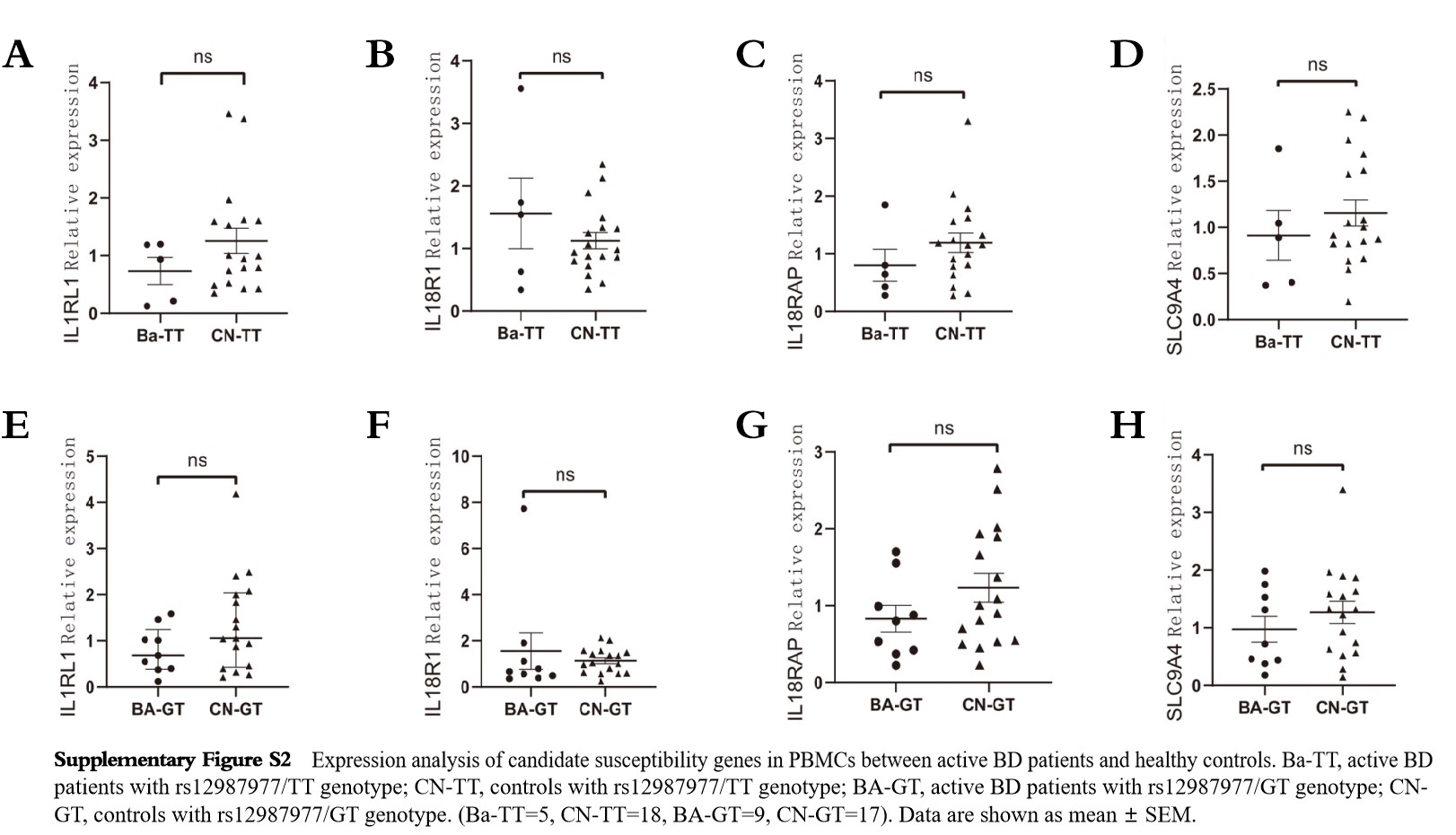

Supplement: Supplementary file 2 [file Image_2.jpg]

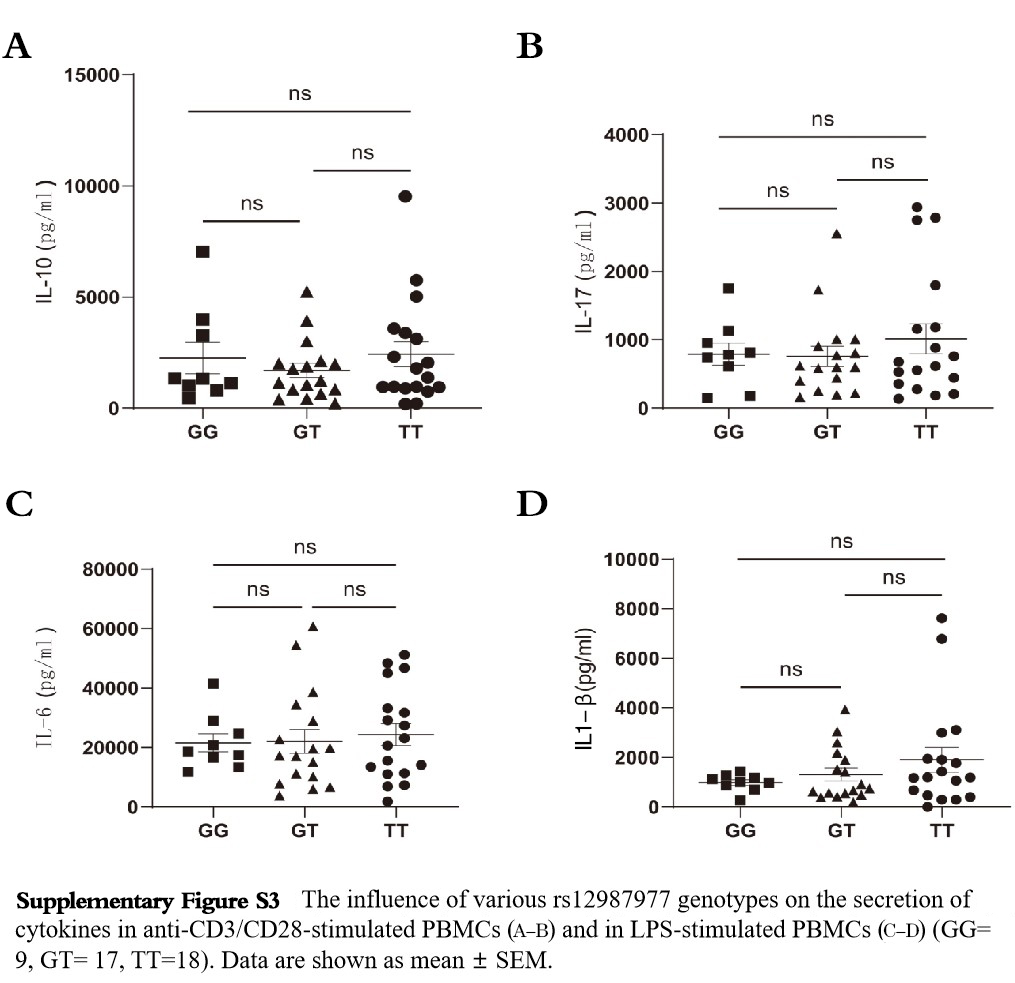

Supplement: Supplementary file 3 [file Image_3.jpg]

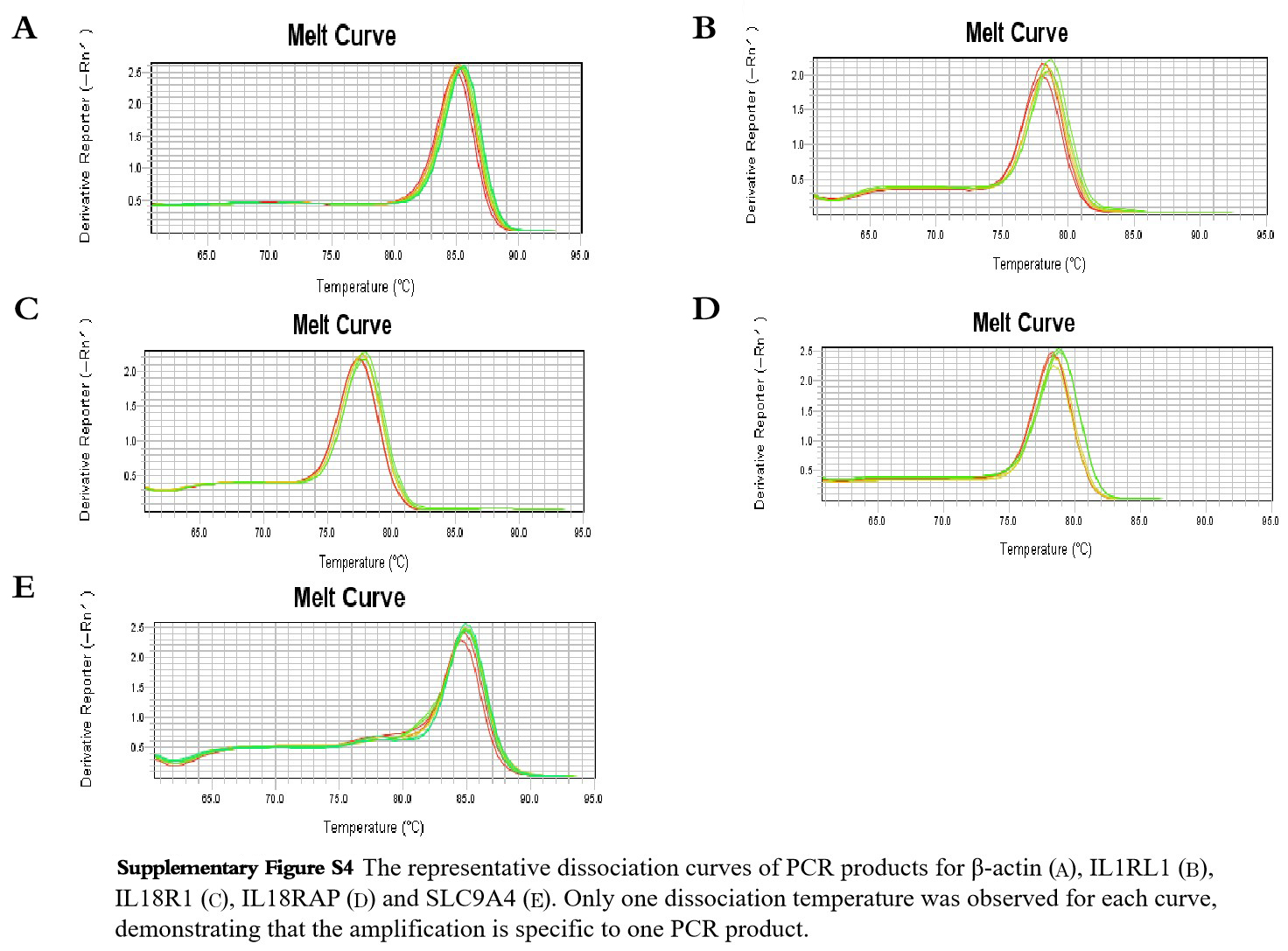

Supplement: Supplementary file 4 [file Image_4.jpg]

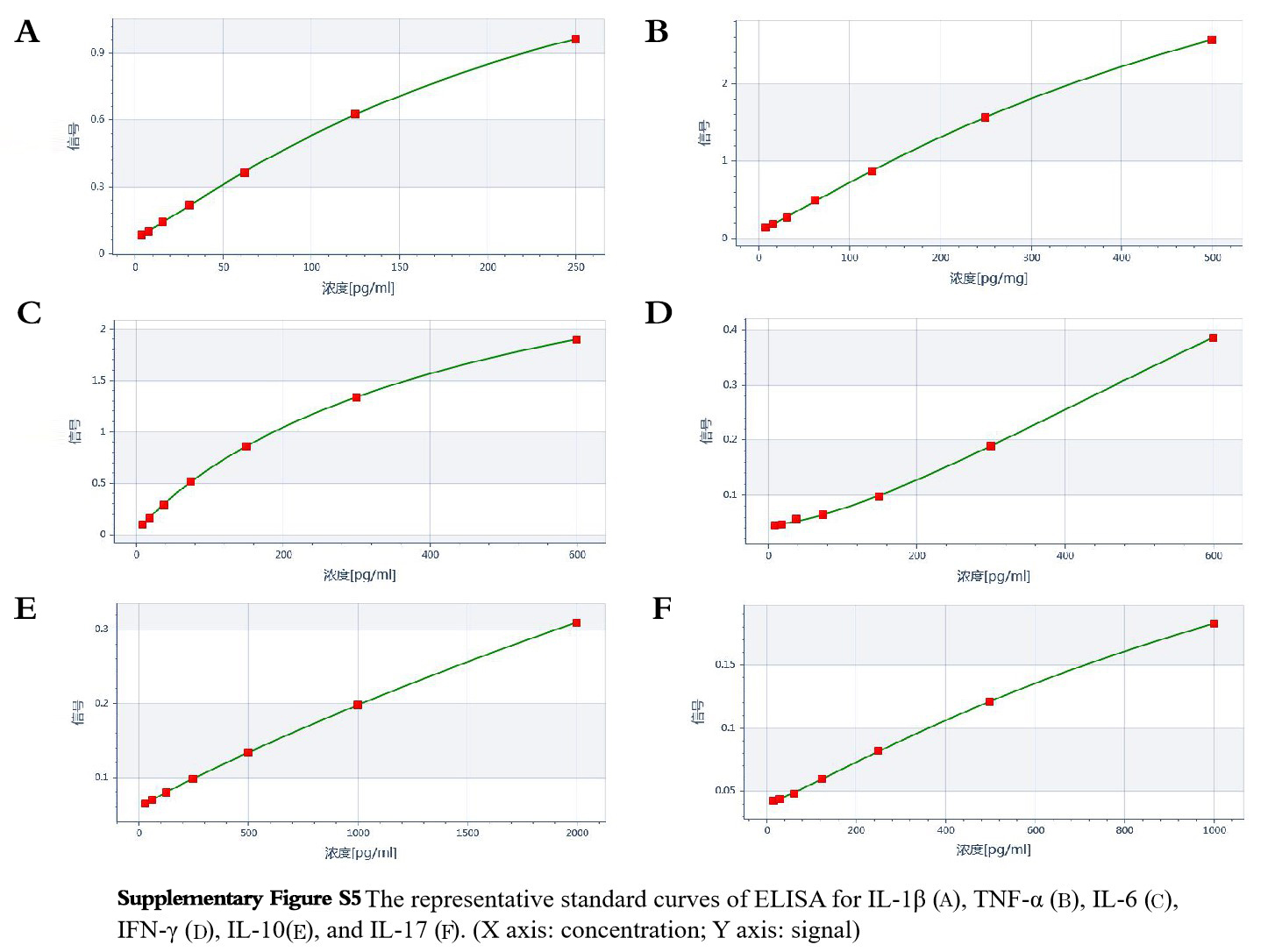

Supplement: Supplementary file 5 [file Image_5.jpg]
