# Supplementary material for: Functional Genetic Polymorphisms in the IL1RL1–IL18R1 Region Confer Risk for Ocular Behçet’s Disease in a Chinese Han Population
Source: Front Genet. 2020 Jul 3;11:645. doi: 10.3389/fgene.2020.00645 (PMC7350896; doi:10.3389/fgene.2020.00645)
Supplement: Supplementary file 6 [file Table_1.docx]

**Supplementary Table S1** Forward and reverse primers for qPCR assays

| **Genes** | **Forward primers** | **Reverse primers** |
| --- | --- | --- |
| β-actin | GAGAAAATCTGGCACCACACC | GGATAGCACAGCCTGGATAGCAA |
| IL1RL1 | TGAGGACGCAGGTGATTACAC | AAAGCCTTGCTCATCCTTGACC |
| IL18R1 | AGGCCACGTCTTCACAAGAG | TGACACACACAGTCACTAGGC |
| IL18RAP | GATTCTGTAGATTCTCCCAGCG | CCTGAGTATCCCCTTCATTTCTGG |
| SLC9A4 | CTGGCACCAAGAATATCCGCTACC | TGTGCCTCTTGCTTCTGAAGTGAC |
