# Supplementary material for: Functional Genetic Polymorphisms in the IL1RL1–IL18R1 Region Confer Risk for Ocular Behçet’s Disease in a Chinese Han Population
Source: Front Genet. 2020 Jul 3;11:645. doi: 10.3389/fgene.2020.00645 (PMC7350896; doi:10.3389/fgene.2020.00645)
Supplement: Supplementary file 7 [file Table_2.docx]

**Supplementary Table S2** Other 6 SNPs in the first-stage association study

| SNPs | Genotype | Case | | Control | | P | Pc | OR (95%CI) |
| --- | --- | --- | --- | --- | --- | --- | --- | --- |
|  |  | N | % | N | % |  |  |  |
| rs2160202 | AA | 29 | 6.7 | 59 | 10.8 | 2.66E-02 | NS | 0.594（0.374-0.945） |
|  | AG | 186 | 43.3 | 248 | 45.6 | 4.67E-01 | NS | 0.910（0.705-1.174） |
|  | GG | 215 | 50.0 | 237 | 43.6 | 4.56E-02 | NS | 1.295（1.005-1.670） |
|  | A | 244 | 28.4 | 366 | 33.6 | 1.28E-02 | NS | 0.781（0.643-0.949） |
|  | G | 616 | 71.6 | 722 | 66.4 | 1.28E-02 | NS | 1.280（1.054-1.554） |
| rs1420106 | GG | 90 | 20.9 | 142 | 25.6 | 8.14E-02 | NS | 0.766（0.567-1.034） |
|  | GA | 215 | 49.9 | 274 | 49.5 | 8.95E-01 | NS | 1.017（0.791-1.308） |
|  | AA | 126 | 29.2 | 138 | 24.9 | 1.28E-01 | NS | 1.245（0.938-1.653） |
|  | G | 395 | 45.8 | 558 | 50.4 | 4.56E-02 | NS | 0.834（0.697-0.997） |
|  | A | 467 | 54.2 | 550 | 49.6 | 4.56E-02 | NS | 1.199（1.004-1.434） |
| rs3755267 | GG | 80 | 18.9 | 141 | 25.5 | 1.49E-02 | NS | 0.682（0.500-0.929） |
|  | GT | 217 | 51.3 | 274 | 49.5 | 5.87E-01 | NS | 1.073（0.833-1.382） |
|  | TT | 126 | 29.8 | 138 | 25.0 | 9.22E-02 | NS | 1.276（0.961-1.694） |
|  | G | 377 | 44.6 | 556 | 50.3 | 1.23E-02 | NS | 0.795（0.664-0.952） |
|  | T | 469 | 55.4 | 550 | 49.7 | 1.23E-02 | NS | 1.258（1.051-1.505） |
| rs6746271 | CC | 92 | 21.3 | 143 | 25.8 | 1.06E-01 | NS | 0.782（0.580-1.054） |
|  | CG | 212 | 49.2 | 275 | 49.5 | 9.10E-01 | NS | 0.986（0.766-1.268） |
|  | GG | 127 | 29.5 | 137 | 24.7 | 9.26E-02 | NS | 1.275（0.960-1.692） |
|  | C | 396 | 45.9 | 561 | 50.5 | 4.26E-02 | NS | 0.832（0.696-0.994） |
|  | G | 466 | 54.1 | 549 | 49.5 | 4.26E-02 | NS | 1.202（1.006-1.437） |
| rs2058660 | AA | 93 | 21.5 | 142 | 25.6 | 1.33E-01 | NS | 0.796（0.591-1.073） |
|  | AG | 211 | 48.9 | 275 | 49.6 | 8.04E-01 | NS | 0.969（0.753-1.246） |
|  | GG | 128 | 29.6 | 137 | 24.7 | 8.50E-02 | NS | 1.282（0.966-1.700） |
|  | A | 397 | 45.9 | 559 | 50.5 | 4.72E-02 | NS | 0.835 (0.699-0.998) |
|  | G | 467 | 54.1 | 549 | 49.5 | 4.72E-02 | NS | 1.198 (1.002-1.431) |
| rs917997 | CC | 88 | 20.6 | 142 | 25.7 | 6.06E-02 | NS | 0.749 (0.554-1.013) |
|  | CT | 214 | 50.0 | 274 | 49.5 | 8.88E-01 | NS | 1.018 (0.791-1.311) |
|  | TT | 126 | 29.4 | 137 | 24.8 | 1.02E-01 | NS | 1.267 (0.954-1.682) |
|  | C | 390 | 45.6 | 558 | 50.5 | 3.15E-02 | NS | 0.822 (0.687-0.983) |
|  | T | 466 | 54.4 | 548 | 49.5 | 3.15E-02 | NS | 1.217 (1.017-1.455) |
